# Intrahepatic cholangiocarcinoma in a transplant liver - selective internal radiation therapy followed by right hemihepatectomy: report of a case

**DOI:** 10.1186/1477-7819-12-198

**Published:** 2014-07-01

**Authors:** Jens Sperling, Christoph Justinger, Jochen Schuld, Christian Ziemann, Roland Seidel, Otto Kollmar

**Affiliations:** 1 Present address: Department of General, Visceral and Pediatric Surgery, University Medical Center Göttingen, Georg August University, D-37075 Göttingen, Germany; 2 Present address: Department of General and Visceral Surgery, Städtisches Klinikum Karlsruhe, D-76133 Karlsruhe, Germany; 3 Department of General, Visceral, Vascular and Pediatric Surgery, University of Saarland, D-66421 Homburg-Saar, Germany; 4 Department of Diagnostic and Interventional Radiology, University of Saarland, D-66421 Homburg-Saar, Germany

**Keywords:** Liver transplantation, Liver resection, Intrahepatic cholangiocarcinoma, Selective internal radiation therapy

## Abstract

Intra- or extrahepatic cholangiocarcinomas are the second most common primary liver malignancies behind hepatocellular carcinoma. Whereas the incidence for intrahepatic cholangiocarcinoma is rising, the occurrence of extrahepatic cholangiocarcinoma is trending downwards. The treatment of choice for intrahepatic cholangiocarcinoma remains liver resection. However, a case of liver resection after selective internal radiation therapy in order to treat a recurrent intrahepatic cholangiocarcinoma in a transplant liver is unknown in the literature so far. Herein, we present a case of a patient undergoing liver transplantation for Wilson’s disease with an accidental finding of an intrahepatic cholangiocarcinoma within the explanted liver. Due to a recurrent intrahepatic cholangiocarcinoma after liver transplantation, a selective internal radiation therapy with yttrium-90 microspheres was performed followed by right hemihepatectomy. Four years later, the patient is tumor-free and in a healthy condition.

## Background

Patients suffering from unresectable intrahepatic cholangiocarcinoma have an overall poor prognosis as demonstrated by a median survival of about three months in untreated patients [1]. In the present case, the patient developed a recurrent intrahepatic cholangiocarcinoma (ICC) in a transplant liver that was initially classified as unresectable. Possible treatment modalities for unresectable ICC are systemic chemotherapy, loco-regional therapies and best supportive care [2]. In selected patients and specialized centers, a neoadjuvant chemotherapy followed by liver transplantation is also feasible [3]. At present, systemic chemotherapy may be seen as the treatment of choice for unresectable ICC [4]. However, a standardized treatment strategy for patients with unresectable ICC has not yet been established. According to the recently updated and published guidelines for the management of ICC, loco-regional therapies can be considered as treatment options in such instances [5]. Selective internal radiation therapy (SIRT) represents a promising loco-regional therapeutic modality for ICC although there are only few data on this issue and no randomized controlled trials until now [6-11]. However, the patient was treated with SIRT and showed good radiological response. In consequence, right hemihepatectomy was performed. Four years later, the patient is tumor free and in a healthy condition with sufficient liver function. The present case report describes the clinical course of the patient in detail and reviews the literature on the management of ICC with SIRT.

## Case presentation

A 55-year-old patient with liver cirrhosis due to Wilson’s disease developed a tumor in segment VI of the liver. Whereas ultrasound-guided biopsy was not effective to achieve a definitive histological diagnosis, the tumor was highly suggestive of a hepatocellular carcinoma (HCC) on computed tomography (CT) and magnetic resonance imaging (MRI). The alpha-fetoprotein serum level was 13.5 IU/ml. The labMELD was 14. The tumor diameter was 18 mm. According to our department standards, HCC was the suspected diagnosis. This was in line with the diagnostic algorithm recently updated and published in the practice guidelines of the AASLD (American Association for the Study of Liver Diseases) [12].

At the time of the suspected diagnosis, the patients Child-Pugh Score was B. Due to the limited liver function combined with a restricted general condition, (ECOG (Eastern Cooperative Oncology Group) Performance Status: 1 to 2), we refrained from a surgical resection. Thus, in line with the BCLC (Barcelona Clinic Liver Cancer classification), the patient was considered for liver transplantation. Moreover, we decided to perform a transarterial chemoembolization (TACE) as a bridging therapy for tumor control. Therefore, the patient underwent four cycles of doxorubicin-based TACE resulting in a stable disease for one year. According to the Milan criteria, the patient was subsequently listed for liver transplantation (LTx).

**Figure 1:**
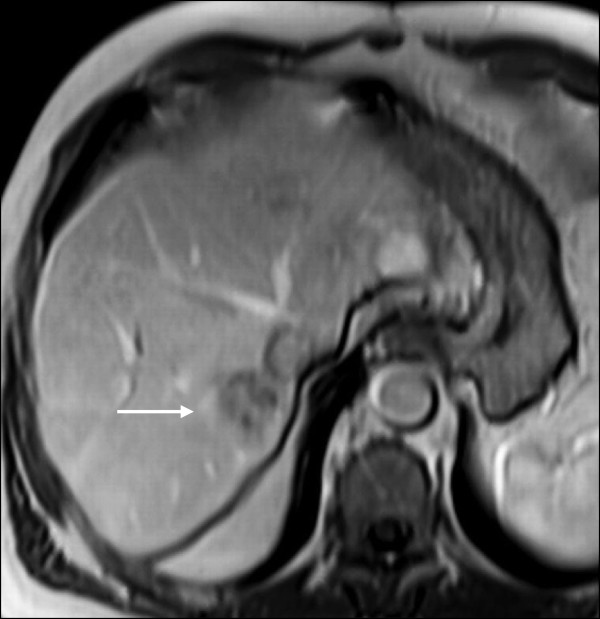
**Magnetic resonance imaging of the transplant liver before selective internal radiation therapy (SIRT).** The arrow points to the ICC in segment VII of the transplant liver.

**Figure 2:**
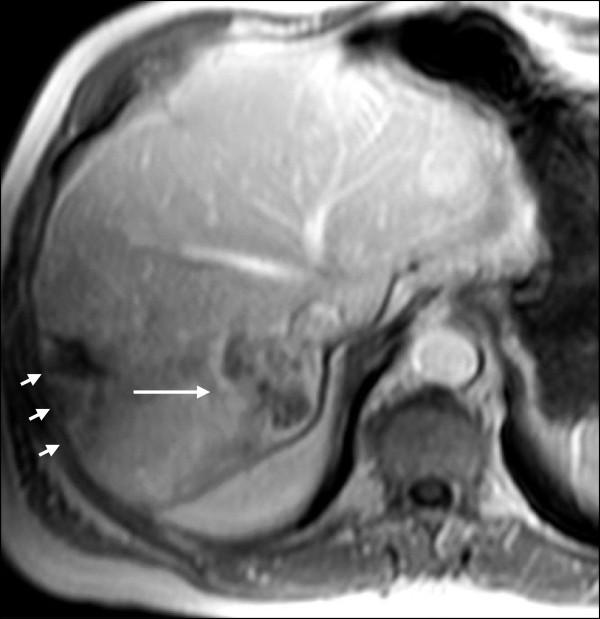
**Magnetic resonance imaging of the transplant liver after selective internal radiation therapy (SIRT).** Note the necrosis within the tumor center of the ICC as indicated by the long arrow. The small arrows point to fibrotic liver alterations due to SIRT.

**Figure 3:**
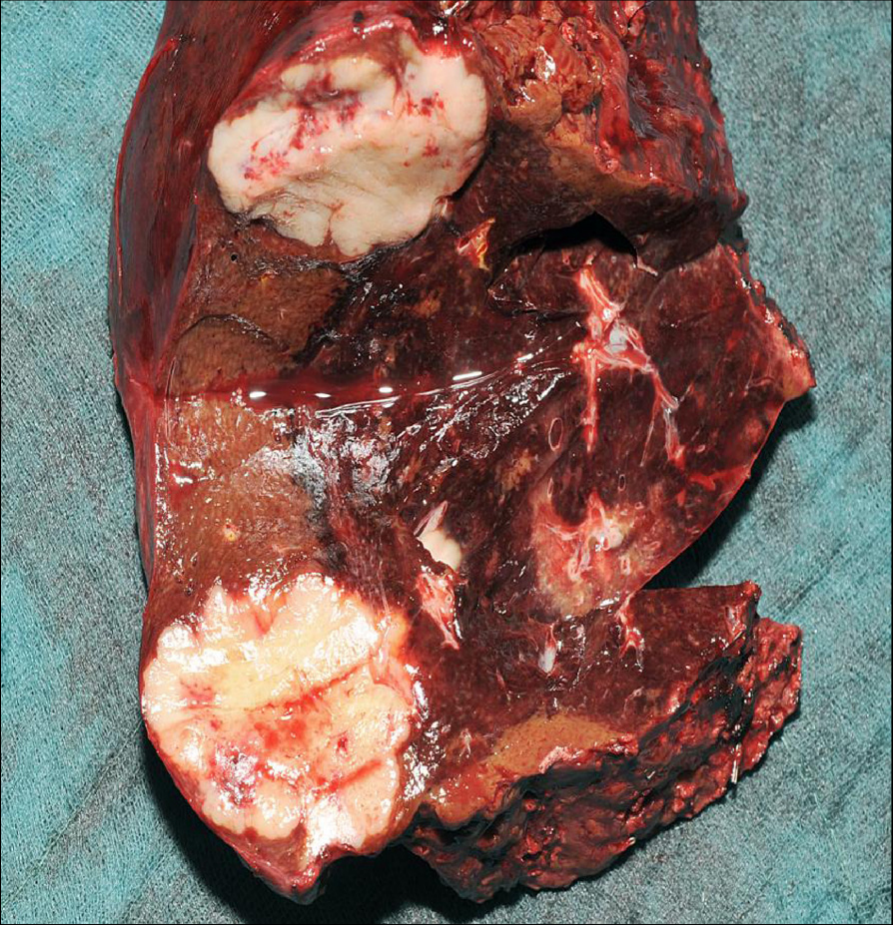
**Macroscopic appearance of the resected specimen.** Note the fibrotic alteration of the adjacent liver.

**Figure 4:**
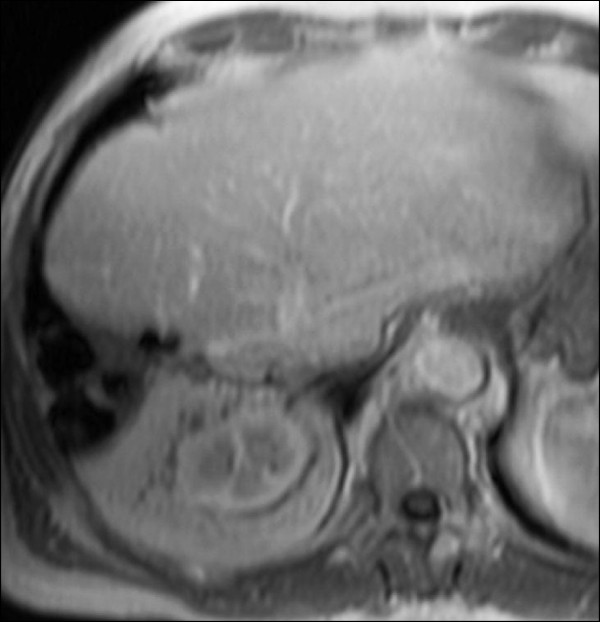
Magnetic resonance imaging of the liver transplant after resection with no evidence of recurrent disease.

Six months later, one and a half years after the initial tumor diagnosis, the patient underwent orthotopic LTx using a venovenous axillofemoral bypass. Initial immunosuppression consisted of cyclosporine, azathioprine and steroids. The peri- and postoperative course was uneventful and the patient was discharged from the hospital on the 15th postoperative day. The tumor in the explanted liver measured 17 × 15 × 14 mm. Surprisingly, the pathological result revealed an ICC (pT1, pN0, GII, R0) instead of the assumed HCC. Two lymph nodes next to the bile duct were free of malignancy. One month after LTx, the patient was admitted to the clinic with a complete fascial dehiscence requiring emergency surgical revision with direct suture. Immunosuppression was converted to tacrolimus monotherapy.Four years after LTx, the patient developed a recurrent ICC in segment VII of the transplant liver in close vicinity to the inferior vena cava (Figure 1). A CT scan revealed no signs of extrahepatic metastases. Immunosuppression was switched to rapamycin. Due to the close vicinity to the vena cava, tumor resection was considered to pose a significant risk for the patient, especially in a transplant liver. The case was intensively discussed by our interdisciplinary tumor board. We decided against tumor resection considering that the ICC was unresectable with respect to its location. Thus, the patient was evaluated for selective internal radiation therapy (SIRT) and underwent a single application of yttrium-90 microspheres (TheraSphere®, Nordion, Ottawa, Canada). On the follow-up MRI scan the tumor showed good radiological response with near total tumor necrosis although with a slight increase in size (Figure 2). Again, the case was intensively discussed by our interdisciplinary tumor board. Due to the promising radiological response but a high risk for tumor cell survival, we offered the patient the possibility of undergoing surgical resection.Due to the tumor location, fibrosis and radiation-associated alteration of the surrounding liver, a right hemihepatectomy was performed using a Cavitron Ultrasonic Surgical Aspirator (CUSA®, Valleylab, Boulder, CO, USA) for parenchymal dissection. Two weeks before liver resection, immunosuppression was switched back to tacrolimus to facilitate wound healing. The tumor size measured 60 × 50 × 42 mm (Figure 3). The histopathological examination showed a large area of necrobiotic change adjacent to 40% of vital tumor cells.The postoperative course was complicated due to biliary fistula from the resection margin, fascial necrosis and symptomatic ileus requiring surgical revision, including the implantation of a Permacol™ biologic implant (Covidien, Mansfield, MA, USA) for definitive abdominal wall closure. Six weeks after the final surgical intervention, the patient was discharged from the hospital in a good general condition with clean wounds. After complete wound healing, immunosuppression was reverted to rapamycin. Two years after resection of the ICC, the transplant liver showed sufficient graft function without signs for tumor recurrence (Figure 4). On the latest follow-up, four years after tumor resection, the patient remains disease-free and in a healthy condition.

This case report is of interest for hepato-biliary and liver transplant surgeons, as it describes the first case of SIRT prior to right hemihepatectomy to treat an ICC within a transplant liver.

Intra- or extrahepatic cholangiocarcinomas are the second most common primary liver malignancies behind HCC [13]. They are relatively rare, although with a rising incidence worldwide [14]. In detail, several international studies have shown that the incidence of ICC is rising, whereas the occurrence of extrahepatic cholangiocarcinoma (ECC) is trending downwards [15]. In case of an ICC, surgical resection remains the only therapy that offers long-term survival or even cure [13]. However, due to bilobular tumor involvement or concomitant liver disease, curative surgery is feasible in only a few patients [13]. In contrast to HCC, for which LTx or TACE are validated treatment options, little evidence of effective alternatives to liver resection exist in the literature for ICCs [16]. TACE is not recommended for this type of tumor as ICCs are mainly fibrotic as well as non-hypervascular and, therefore, not the ideal target for this treatment modality. However, in the present case the tumor was primarily treated with four cycles of TACE assuming a HCC within the cirrhotic liver. Due to a limited liver function we decided against tumor resection. Although resection can be performed in patients with advanced liver disease, mortality is probably higher and patients might be better served by liver transplantation or ablation [12]. However, we do not perform a radiofrequency ablation but TACE. This therapy form was chosen for tumor control in terms of a bridging therapy until liver transplantation. Of interest, the tumor remained stable for one year.

Whereas LTx is well established for HCC [17] it is far from being a well accepted indication for ICC or ECC. Regarding ECC, there are, beside careful patient selection, well defined neoadjuvant protocols prior to LTx which have led to encouraging results for this tumor entity [18-23]. However, the role of LTx in the management of ICC still remains controversial because of organ shortage, disease recurrence and the risk of accelerating tumor progression under immunosuppression after transplantation [13]. Farges *et al*. even claim that there are only two exceptions where LTx for ICC lead to good results: first, LTx for very small ICCs that have been mistaken for HCC (like in our case) or incidentally discovered ICC within the explanted liver [16], and second, ECC from second order biliary branches, which can be viewed as ICC and treated by an aggressive neoadjuvant regimen [16]. However, the key determinant identified for a promising outcome is a low tumor stage [24,25]. ICCs with lymph node metastasis, vascular or bile duct invasion remain contraindications for LTx [25].

In the present case, the patient underwent LTx with an ICC in segment VI that was mistakenly diagnosed as HCC. Our patient developed recurrent disease four years after LTx, which is considered to be a very long time period of tumor free survival. The recurrent tumor was initially considered to be unresectable. Systemic chemotherapy may be seen as the treatment of choice in such instances with, however, palliative intent [4]. To date, a treatment strategy for these patients has not been standardized. Beside systemic chemotherapy, loco-regional therapies can be performed in patients suffering from non-resectable disease [5]. At present there are no established first-line loco-regional therapeutic options for patients with unresectable ICC [5].

SIRT, however, represents a promising treatment modality although there are only few data on it and no randomized controlled trials [6-11].

SIRT is a radioembolization procedure which is considered to be an effective liver-directed therapy with a favorable therapeutic ratio that offers meaningful benefits for selected patients [26,27]. SIRT is able to improve survival after intrahepatic recurrence of ICC [10]. Of interest, the antitumor effect of SIRT is rather related to radiation than to embolization [28]. Thereby, it provides a low toxicity profile and is able to deliver extremely high doses up to small target volumes while sparing the surrounding liver tissue [29]. It remains debatable if the clinical situation in the present case is comparable to a recurrent ICC in a non-transplanted liver. However, after discussing the case in our interdisciplinary tumor board meetings, we decided to treat the patient with SIRT assuming that he was in an overall non-curative situation. SIRT can be conducted to downsize the intrahepatic tumor mass, because it is known that the use of yttrium-90 microspheres is useful for this purpose and might make the surgical resection easier [30]. In the present case, SIRT led to relevant tumor necrosis as demonstrated by good radiological response. Thus, we decided to offer the patient surgical resection. However, due to relevant fibrosis combined with radiation-associated alteration induced by SIRT, right hemihepatectomy was needed while initially a limited resection may have been possible. To the best of our knowledge no similar case has been reported in the literature so far.

Another important issue in dealing with liver resection in a transplant liver is immunosuppression. The mTOR (mammalian target of rapamycin)-inhibitor rapamycin is known to inhibit tumor growth *in vitro* and *in vivo*[31-34]. Therefore, this drug provides the advantage of combining immunosuppression with anticancer therapy. The most important side effect of rapamycin is its negative influence on wound healing caused by its antiangiogenetic component [35]. In the present case, the rapamycin-induced-immunosuppression was switched back to tacrolimus two weeks before liver resection to facilitate wound healing.

Nevertheless, the patient developed a biliary fistula combined with fascial necrosis. It may be that these complications might have been preventable if the mTOR-inhibitor had been stopped earlier. From our experiences it could, therefore, be recommended for repeated surgery in LTx-patients under mTOR-inhibition, that immunosuppression should be switched to tacrolimus or cyclosporine at least four weeks before surgery. After complete wound healing, the immunosuppression could then be reverted to mTOR-inhibition.

In addition, abdominal wall hernia is one of the most common surgical problems after LTx with an incidence ranging between 4.6 and 23% [36,37]. Hernia repair with synthetic mesh graft showed good results, although these grafts have a high risk of infection leading to postoperative wound healing complications [38,39]. In the present case, we successfully used a Permacol™ implant for the infected hernia side classified grade 3 to 4 according to the Ventral Hernia Working Group [40].

## Conclusion

In conclusion, we demonstrated for the first time a multimodal treatment approach for ICC, including LTx for the primary ICC and SIRT prior to liver resection for recurrent/*de novo* ICC, with encouraging results. Furthermore, we were able to show that an abdominal wall hernia in an infected hernia site after LTx can be successfully repaired with a Permacol® biologic implant.

## Consent

Written informed consent was obtained from the patient for publication of this Case Report and any accompanying images. A copy of the written consent is available for review by the Editor-in-Chief of this journal.

## Abbreviations

AASLD: American Association for the Study of Liver Diseases; BCLC: Barcelona Clinic Liver Cancer classification; CT: computed tomography; ECC: extrahepatic cholangiocarcinoma; ECOG: Eastern Cooperative Oncology Group; HCC: hepatocellular carcinoma; ICC: intrahepatic cholangiocarcinoma; Ltx: liver transplantation; MRI: magnetic resonance imaging; mTOR: mammalian target of rapamycin; SIRT: selective internal radiation therapy; TACE: transarterial chemoembolization.

## Competing interests

All authors declare that they have no competing interests.

## Authors’ contributions

J Sperling, J Schuld and OK performed the surgery; RS performed the selective internal radiation therapy; CJ and CZ helped collecting references and post-operation management; JS and OK drafted the manuscript. All authors read and approved the final manuscript.
